# Probenecid Inhibits NLRP3 Inflammasome Activity and Mitogen-Activated Protein Kinases (MAPKs)

**DOI:** 10.3390/biom15040511

**Published:** 2025-04-01

**Authors:** Les P. Jones, David E. Martin, Jackelyn Murray, Fred Sancilio, Ralph A. Tripp

**Affiliations:** 1 Department of Infectious Diseases, University of Georgia, Athens, GA 30602, USA; lj66@uga.edu (L.P.J.); jcrab@uga.edu (J.M.); 2 TrippBio, Inc., Jacksonville, FL 32256, USA; davidmartin@trippbio.com; 3 Department of Chemistry and Biochemistry, Florida Atlantic University, Jupiter, FL 33431, USA; fredsancilio@clearwayglobal.com

**Keywords:** antiviral, anti-inflammatory, host-directed, inflammasome, J774A.1 mouse macrophages

## Abstract

Probenecid has long been a versatile drug in pharmacological therapies, primarily known for blocking active tubular secretion in the kidney, affecting both endogenous substances like uric acid and exogenous ones like penicillin. Beyond its renal applications, probenecid has shown capabilities in crossing the blood–brain barrier and modulating the activity of various membrane channels and transporters. This compound has emerged as a potent antiviral agent, demonstrating efficacy against multiple viruses, including influenza, COVID-19, and RSV. Clinical trials with COVID-19 patients have confirmed its antiviral potential, sparking further investigation into its mechanisms of action. This study explores probenecid’s significant anti-inflammatory properties, focusing on its ability to inhibit inflammasome activation. Our study aims to unravel the anti-inflammatory effects of probenecid on the NLRP3 inflammasome and MAPK signaling pathways using murine macrophages as a relevant inflammation model. We reveal that probenecid treatment blocks JNK and ERK signaling without affecting p38 MAPK, suppressing NLRP3 inflammasome activation. Additionally, probenecid does not affect NFκB-directed protein expression, although it efficiently inhibits NLRP3 inflammasome outputs, e.g., IL-1β and pyroptosis. These results indicate probenecid’s potential therapeutic applications.

## 1. Introduction

Respiratory virus infections cause inflammatory and immune responses. These responses are defensive mechanisms aimed at reducing illness and disease. There is a need for safe and effective antiviral and anti-inflammatory drugs to treat respiratory viral infections. Our laboratory is focused on discovering antiviral drugs that prevent viral replication associated with disease and inflammation. We have shown that probenecid, a uricosuric agent used to treat gout and reduce the renal excretion of antibiotics, has broad antiviral and anti-inflammatory properties [1,2,3,4,5]. As an antiviral drug, nanomolar to micromolar treatment with probenecid has been shown to inhibit SARS-CoV-2 variants, influenza virus A strains, human metapneumovirus (HMPV), and RSV A and B strain replication in vitro and in vivo, and demonstrate antiviral activities in a Phase 2 SARS-CoV-2 study [2,5,6,7,8,9]. Specifically, probenecid has been shown to inhibit the replication of ancestral SARS-CoV-2 and variants (Beta, Gamma, Delta, and Omicron, B.1.1) in Vero E6 cells and NHBE cells, as well as to inhibit the replication of RSV and influenza A strains in several respiratory epithelial cell lines, e.g., A549 cells, Calu-3 cells, and normal human bronchoepithelial (NHBE) cells. This broad antiviral activity indicates that probenecid affects virus replication by a common host cell pathway used by more than one virus for replication. Recently, we showed that probenecid regulates the conserved mitogen-activated protein kinase (MAPK) pathways used for virus replication, specifically, extracellular signal-regulated kinases (ERKs) and c-Jun N-terminal kinases (JNKs) affecting virus replication [1]. The MAPK pathway is a signaling cascade that transduces extracellular signals, including virus infection [10].

Probenecid has been shown to modify ACE2 expression in normal human lung tissue [11], the expression of the pannexin 1 (PANX1) gene regulating inflammation [12,13,14], and the host responses to virus infection [2,4,8,15]. Studies have shown that PANX1 signaling exacerbates inflammatory reactions by secreting proinflammatory cytokines such as IL-1β and IL-6 [12]. Additionally, IL-1β and IL-6 are essential in the activation of fever through the hypothalamic–pituitary–adrenal axis and the induction of eicosanoids [13,14]. Inflammasomes are protein complexes assembled following cellular infection or stress such as that caused by viral infection [15]. Their activation is a functional measure of inflammatory activation as they regulate the maturation of the pro-inflammatory cytokines such as IL-1β, leading to inflammation and modified immune defense [16]. Probenecid has been shown to inhibit the inflammasome response primarily by blocking the PANX1 channel, which has a crucial role in inflammasome activation, thereby reducing the production of pro-inflammatory cytokines like IL-1β and IL-18 [17]. One notable inflammasome is NLRP3 (NOD- LRR- and pyrin domain-containing protein 3), an intracellular sensor that detects various microbial motifs, danger signals, and environmental irritants. Assembly of the NLRP3 inflammasome leads to caspase 1 (cas-1)-dependent release of the pro-inflammatory cytokines IL-1β and IL-18, as well as to gasdermin D (GASD)-mediated pyroptotic cell death [18]. NLRP3 inflammasome responses promote and may contribute to disease severity, and inhibiting NLPR3-mediated inflammation may reduce inflammation [19]. Understanding the activation of NLRP3 inflammasomes and airway inflammatory diseases has advanced the development of drugs that specifically inhibit the activation of the inflammasome complex by targeting its components like NLRP3 and cas-1 or the downstream expression of IL-1β or IL-18 to treat inflammatory diseases associated with its overactivation, which are being investigated [20]. Different types of inflammasomes, e.g., NLRC4, NLRP1, and NLRP6, have been identified [21]. NLRP3 recruits pro-cas-1 by specific interaction of the pro-cas-1 CARD domain with the CARD domain on the ASC adaptor protein linked to NLRP3 through their common pyrin domains and cleaves it to cas-1, which processes pro-IL-1β to IL-1β [22]. Cas-1 also processes GASD, a protein required for pyroptosis and the secretion of IL-1β in both canonical and non-canonical inflammasome responses [23]. Pyroptosis is a type of programmed cell death characterized by inflammation and the release of cellular contents mediated by GASD, which forms pores within the plasma membrane [24] to promote cytolysis and the release of interleukin-1 family cytokines into the extracellular space [25]. Probenecid can inhibit the NLRP3 inflammasome response mediated through the P2X7 receptor to reduce the hyperinflammation associated with severe influenza virus infection [26]. Interestingly, probenecid has been shown to reduce inflammation mediated through the P2X7 receptor and increase bacterial clearance in a murine model of *P. aeruginosa* pneumonia without a direct antibacterial effect [27], suggesting that probenecid can reduce inflammation in the absence of antiviral function. It is proposed that opening a non-specific hemichannel formed by pannexin 1 after P2X7 receptor ligation by ATP is necessary for NLRP3 activation [28]. However, studies using differentiated murine BMDMs genetically ablated for Panx1 (-/-) showed that pannexin 1 is dispensable for canonical or noncanonical NLRP3 inflammasome activation [29]. Studies have shown that although diverse NLRP3 activators, including bacterial pore-forming toxins, nigericin, ATP, and particulate matter, can instigate the opening of membrane pores, this is not necessary for NLRP3 activation. Instead, NLRP3 agonists typically mediate the reduction in intracellular potassium, which is both essential and sufficient for caspase-1 activation [18].

Nigericin, a polyether ionophore that exchanges potassium and hydrogen ions across cell membranes [30], was used in this study to activate NLRP3 inflammasomes after LPS priming of J774A.1 macrophages. Nigericin treatment, specifically, anti-ports potassium ions in exchange for protons. The depletion of cytosolic potassium ions induces the oligomerization of NLRP3 inflammasome, activating cas-1 [31]. Nigericin is considered an exclusive NLRP3 agonist in hematopoietic cells, as genetic knockout and chemical inhibition studies on NLRP3 reveal the total inhibition of nigericin-dependent inflammasome activity in murine macrophages. In contrast, other inflammasome sensors, such as NLRC4, AIM2, and MEFV (a human gene involved in making pyrin that has a role in inflammation and infection [32]), do not respond to nigericin [33]. As a result, nigericin-dependent inflammasome activity is considered diagnostic for NLRP3 functionality, specifically in the J774A.1 macrophage, which facilitates quantifying NLRP3 inflammasome components such as IL-1β and TNFα released into the macrophage supernatants post-stimulation. Thus, we examined the IL-1β and TNFα levels in J774A.1 macrophage supernatant following LPS and nigericin stimulation to gauge NLRP3 activation. We also treated these macrophages with MAPK inhibitors of each of the three major MAPK pathways (ERK, JNK, and p38) to determine the effects of MAPKs on NLRP3 inflammasome activation. The results showed that JNK signaling is required for NLRP3 inflammasome activation and that probenecid treatment inhibits ERK and JNK signaling but not p38. Specifically, the results show that probenecid inhibits IL-1β maturation but does not affect TNFα production after NLRP3 inflammasome activation in J774A.1 macrophage. These results show that probenecid treatment is associated with losing the catalytically active p20 subunit of cas-1, indicating that probenecid acts to abrogate cas-1 auto-proteolysis of NLRP3 inflammasomes. Additionally, probenecid treatment inhibits pyroptosis, and cas-1-dependent GASD is required for pore formation in the plasma membrane in NLPR3 inflammasome-activated macrophages. Additionally, the results show that probenecid treatment does not affect the NF-κB-dependent expression of pro-cas-1, pro-GASD, and pro-IL-1β proteins. Taken together, these findings suggest that probenecid acts to inhibit the processing of pro-cas-1 to catalytically active cas-1, and as active cas-1 is necessary for the maturation of pro-IL-1β and pro-GASD substrates, probenecid inhibits the processing of cas-1 and cas-1 substrates, i.e., IL-1β and GASD.

## 2. Materials and Methods

### 2.1. Cell Culture and Drug Treatment

J774A.1 murine macrophages (ATCC TIB-67; isolated in 1968 from the ascites of an adult female mouse with reticulum cell sarcoma) were propagated in DMEM (Gibco, Waltham, MA, USA) + 10% heat-inactivated FBS (Hyclone, Logan, UT, USA) at 37 °C. The cell media were changed to complete DMEM containing the drug treatments as appropriate. Probenecid, ERK inhibitor (PD98059), JNK inhibitor (SP600125), p38 inhibitor (SB202190), and NLRP3 inflammasome inhibitor (MCC950) were diluted in a 0.1% DMSO solution and added at the indicated concentrations to the macrophages and cultured at 37 °C. The MAPK inhibitors, SP600125 [34], PD98059 [35], and SB202190 [36], are selective and biologically relevant, e.g., for inhibition of viral replication [1,37] at the concentrations tested. The NLRP3 inflammasome inhibitor MCC950 was previously shown to inhibit pyroptosis at the concentration tested [38].

### 2.2. Cell Lysates and Western Blots

The culture supernatants were removed, adjusted with 2 mM PMSF (Millipore Sigma, Burlington, MA, USA) protease inhibitor, and stored at −80 °C until use. The treated J774A.1 macrophages were washed twice with ice-cold 1× PBS, and the total protein was dissolved in equal volumes of 1× Laemmli buffer for SDS-PAGE and Western blotting. Western blotting of whole-cell lysates was conducted to detect the phospho-ERK1/2, phospho-JNK1/2, phospho-p38, NLRP3, and phospho-IκBα proteins. Western blots for detecting pro-cas-1, mature cas-1 (p20 subunit), pro-GASD, mature GASD (N-terminal fragment), and pro-IL-1β used a 1:1 mixture of whole-cell lysate and conditioned culture supernatant. Equal amounts of cell lysates (20 μL) or 1:1 mixtures of cell lysate and conditioned culture supernatant (20 μL) were separated on 4–20% gradient SDS-PAGE gels (BioRad, Hercules, CA, USA) and then transferred to nitrocellulose membrane (BioRad) for immunoprobing. The membranes were blocked in Tris-buffered pH 7.5 saline solution containing 0.05% Tween-20 (Millipore Sigma, Burlington, MA, USA), (TBS-T), and 5% BSA for 1 h at room temperature and then incubated with the primary antibody in TBS-T overnight at 4 °C. Specifically, anti-phospho-p44/42 MAPK (ERK1/2) (Thr202/Tyr204) rabbit monoclonal antibody (mAb), anti-phospho-SAPK/JNK (Thr183/Tyr185) rabbit mAb, anti-phospho-p38 MAPK (Thr180/Tyr182) rabbit mAb (Cell Signaling, Danvers, MA, USA), anti-NLRP3 rabbit mAb (Cell Signaling), anti-phospho-IκBα mAb (Thermo Fisher, Waltham, MA, USA), anti-cas-1 rabbit mAb (Cell Signaling), anti-cleaved cas-1 (Asp296) rabbit mAb (Cell Signaling), anti-GASD rabbit mAb (Cell Signaling), anti-cleaved GASD (Asp276) rabbit mAb (Cell Signaling), and anti-cas-1 (p20) mAb (Casper-1) (Adipogen Biosciences, San Diego, CA, USA) were used. After three washes in TBS-T, the membranes were incubated with mouse anti-rabbit IgG (H + L) or goat anti-mouse IgG (H + L), cross-adsorbed secondary antibodies, and HRP conjugates (Thermo Fisher, Waltham, MA, USA) in TBS-T for 2 h at room temperature. The membranes were washed three times with TBS-T, and the signal was developed using the SuperSignal West Pico PLUS chemiluminescent substrate (Thermo Fisher, Waltham, MA, USA). We used the no-stain protein labeling reagent (Thermo Fisher, Waltham, MA, USA, A44717) to normalize protein loading and quantitate the target protein bands in Western blots [39]. The iBright 1500 Imager (Thermo Fisher, Waltham, MA, USA) software performs densitometry on the no-stain fluorescence signal from each lane’s total protein and generates a normalization factor based on the total protein signal. The software then adjusts the densitometric signal from the target proteins based on the normalization factors. Total protein staining of the transferred membranes was used to normalize for protein loading with (n = 3) independent replicates for each condition. Immunoblot and total protein images were obtained using the iBright 1500 Imager (Thermo Fisher, Waltham, MA, USA), representing three independent replicates per condition. Background-corrected band volumes were normalized to total protein, and the mean was calculated from three independent replicates for each condition using iBright analysis software (Thermo Fisher, Waltham, MA, USA). The graphs show absolute band volume quantification in arbitrary units (A.U.) represented as mean ± SD and the fold change in mean band volume as mean ± SEM relative to the diluent treatment condition.

### 2.3. Canonical NLRP3 Inflammasome Activation

The J774A.1 macrophages were plated at 4 × 10^5^ cells/well in 24-well plates and cultured overnight in complete DMEM at 37 °C. The media were replaced with fresh DMEM containing 100 ng/mL LPS from *E. coli* O111:B4 (Millipore Sigma, Burlington, MA, USA) for 6 h; then, 20 μM nigericin (R&D Systems, Minneapolis, MN, USA) was added for 1 h, LPS was added for 6 h, or probenecid or inhibitors were added for the last 2 h of the LPS treatment before the nigericin addition. The culture supernatants were harvested, supplemented with 2 mM PMSF protease inhibitor (Millipore Sigma, Burlington, MA, USA), and stored at −80 °C until use.

### 2.4. IL-1β, TNFα, and LDH Detection

The IL-1β and TNFα levels in the J774A.1 macrophage cell culture supernatant were assessed using the DuoSet ELISA Kit (R&D Systems, Minneapolis, MN, USA), a mouse-specific IL-1β or TNFα ELISA assay. Per the manufacturer’s instructions, LDH in the cell culture supernatant was assayed using the CytoxTox 96 non-radioactive cytotoxicity assay (Promega, Madison, WI, USA). The data are expressed as a percentage of maximal release.

### 2.5. Chemicals Used

LPS from *E. coli* O111:B4, SP600125 JNK inhibitor, SB202190 p38 inhibitor, PD98059 ERK inhibitor, probenecid, MCC950 NLRP3 inhibitor, DMSO, and PMSF was obtained from MilliporeSigma, Burlington, MA, USA. Nigericin was obtained from R&D Systems, Minneapolis, MN, USA.

### 2.6. Statistical Analysis

GraphPad Prism 10 software was used to perform all the statistical analyses. Differences between groups were analyzed using multiple unpaired *t*-tests with Welch’s correction applied, and *p* values are indicated in the graphs. Data are reported as mean ± standard deviation (SD). The error associated with the fold-change data was determined by calculating the standard error of the mean (SEM) for both the control and experimental groups and then utilizing error propagation to determine the SEM of the fold-change value.

## 3. Results

**Figure 1:**
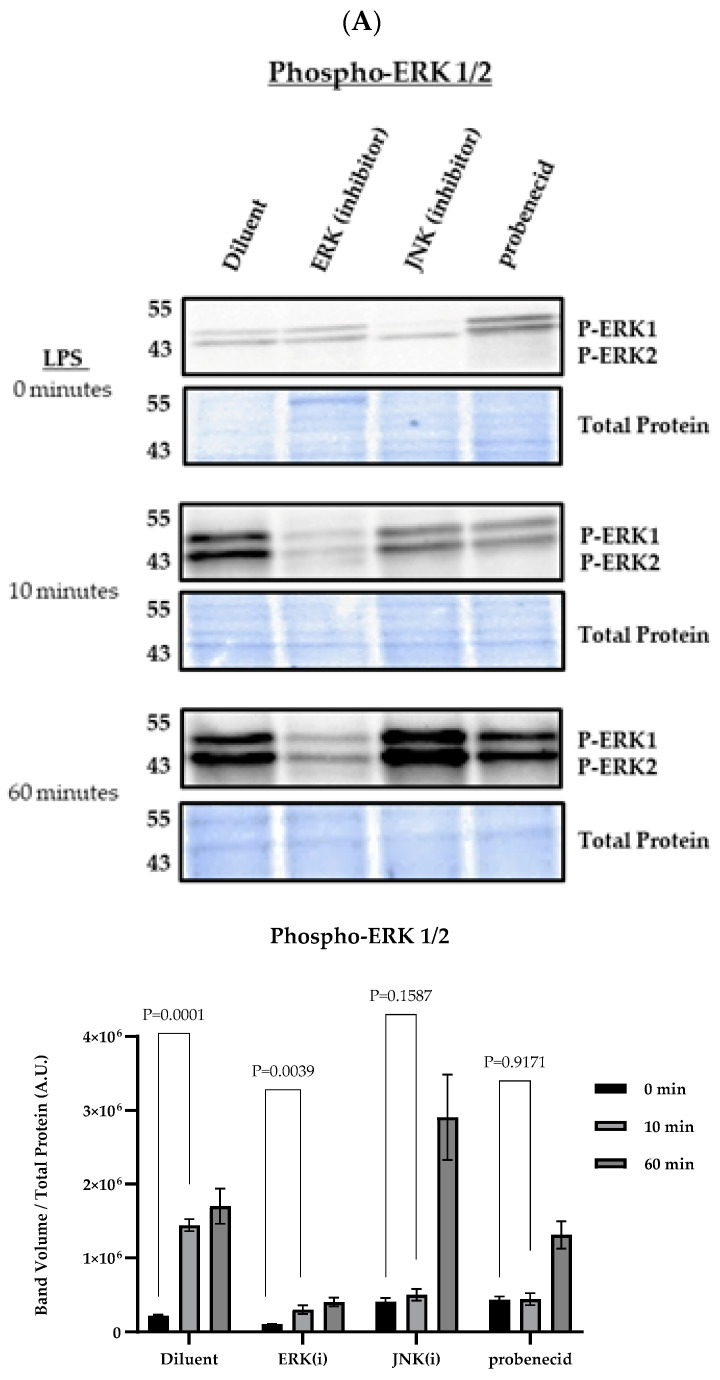

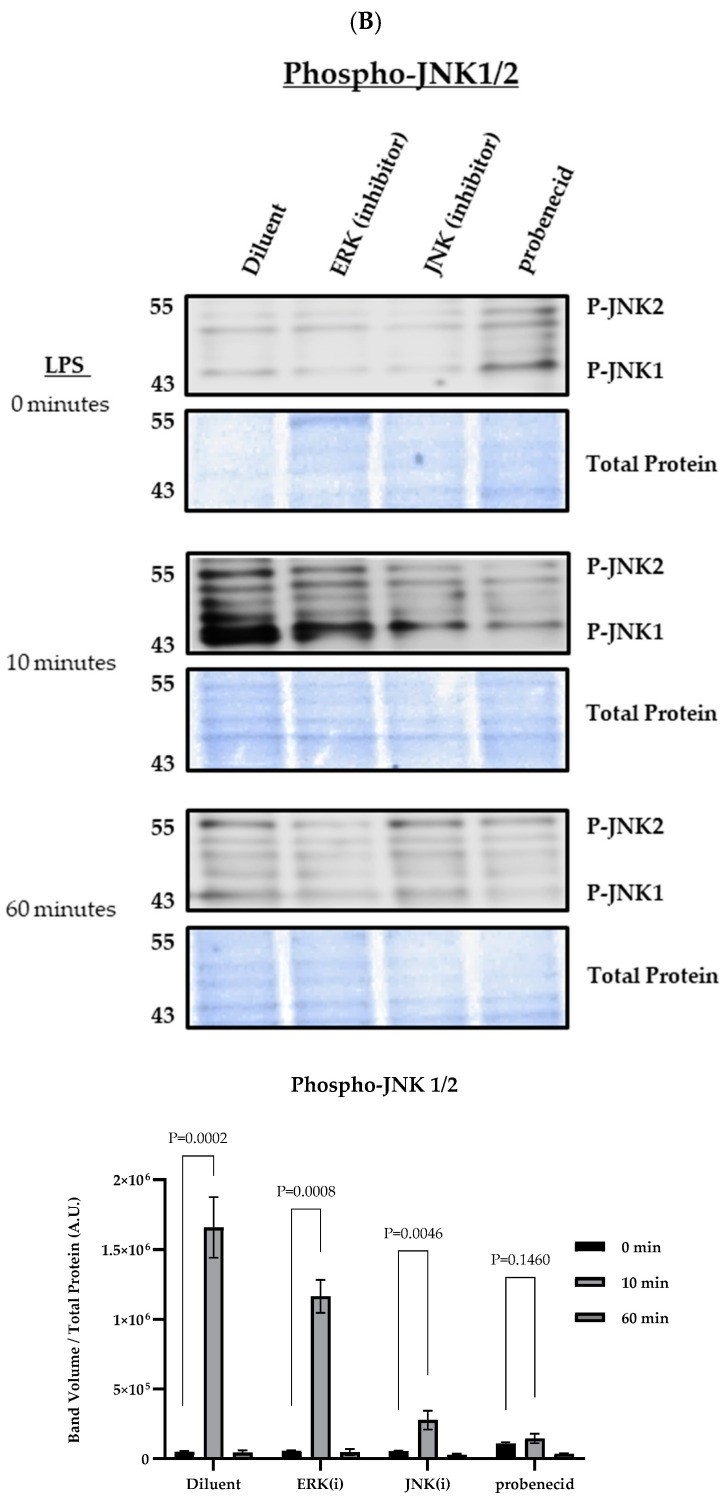

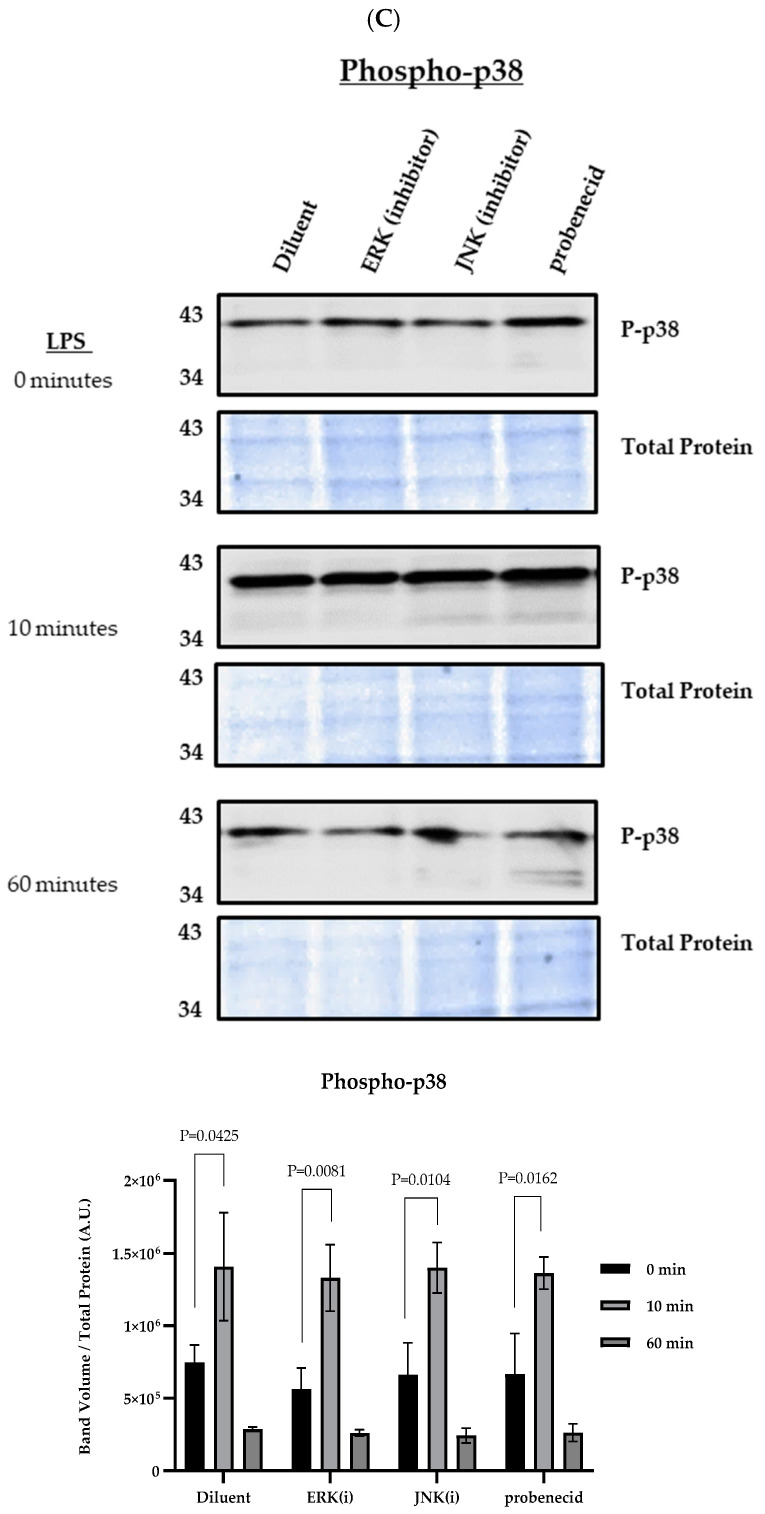
MAPK signaling in LPS-primed macrophages. J774A.1 macrophages were treated with 0.1 µM probenecid, 25 µM JNK inhibitor (SP600125), 10 µM ERK inhibitor (PD98059), or diluent (0.1% DMSO) as described. (**A**) Samples were probed for phospho-ERK1/2 proteins using anti-phospho-p44/42 MAPK (ERK1/2) (Thr202/Tyr204), (**B**) phospho-JNK1/2 proteins using anti-phospho-SAPK/JNK (Thr183/Tyr185), or (**C**) phospho-p38 proteins using anti-phospho-p38 MAPK (Thr180/Tyr182) (Cell Signaling). The membranes were washed three times with TBS-T, and the signal developed with SuperSignal West Pico PLUS chemiluminescent substrate (Thermo Fisher). Total protein staining of transferred membranes utilizing the fluorescent ‘no-stain’ protein labeling reagent (Thermo Fisher) was used to normalize for protein loading with (n = 3) independent replicates for each condition. Immunoblot and total protein blot images were obtained using the iBright 1500 Imager (Thermo Fisher), representing three independent replicates per condition. Background-corrected band volumes were normalized to total protein, and the mean was calculated from three independent replicates for each condition using iBright analysis software (Thermo Fisher). The graphs depict the band volume quantification in arbitrary units (A.U.) represented as mean ± SD. Original western blots can be found at Supplementary Materials.

To determine the effect of the probenecid on the MAPK signaling and NLRP3 inflammasome activity, the J774A.1 macrophages were treated with 0.1 μM probenecid and then primed with 100 ng/mL LPS from *E. coli* (0111: B4). LPS is a TLR4 agonist that induces MAPK signaling [40]. We used 0.1 μM probenecid in all the studies as that concentration best inhibited IL-1β in the J774A.1 macrophage supernatant from the activated cells. It is known that NFκB activation and TNFα release are required to initiate the first signal in potentiating NLRP3 inflammasome activation [41]. The macrophages were treated for 2 h with 0.1 μM probenecid, or 10 μM MAP kinase kinase (ERK inhibitor; PD98059), or 25 μM JNK inhibitor (SP600125), or diluent (0.1% DMSO; Figure 1), and then stimulated with 100 ng/mL LPS. The results show that probenecid treatment inhibits the induction of phospho-ERK by nearly 50% and phospho-JNK by >90% at 10 min post-LPS stimulation compared to diluent-treated macrophages, whereby phospho-ERK was induced by >5-fold, and phospho-JNK was induced 35-fold (Figure 1A,B). As expected, the ERK inhibitor (10 μM PD98059) reduced phospho-ERK levels by 80% at 10 min post-LPS, and at 60 min, there was a >50% reduction compared to the diluent. In contrast, the inhibitory effects of probenecid treatment on phospho-ERK were <20% compared to the diluent (Figure 1A). Notably, the JNK inhibitor (SP600125) reduced phospho-ERK by 50% at 10 min post-LPS stimulation compared to the diluent. In comparison, phospho-ERK was increased by 40% at 60 min post-LPS stimulation in the presence of the JNK inhibitor compared to the diluent (Figure 1A). These results suggest that cross-talk and signal compensation mechanisms occur between the MAPK signaling cascades, as predicted by other studies [42,43]. At 10 min post-LPS stimulation, the 25 μM SP600125 treatment reduced phospho-JNK by approximately 90% compared to the robust JNK phospho-activation, i.e., a 35-fold induction (Figure 1B). the probenecid treatment inhibited phospho-JNK at 10 min post-LPS stimulation by >90% compared to the diluent-treated macrophages. Phospho-JNK was minimally detectable after 60 min of LPS stimulation (Figure 1B). This finding is consistent with studies examining JNK signaling following LPS treatment in murine macrophages [44]. The effect of probenecid treatment on phospho-p38 was also determined in LPS-treated macrophages (Figure 1C). The results suggest that p38 signaling in response to LPS stimulation is not affected by probenecid treatment, ERK inhibitor (10 μM PD98059), or JNK inhibitor (25 μM SP600125) compared to the diluent-treated macrophages (Figure 1C).

**Figure 2:**
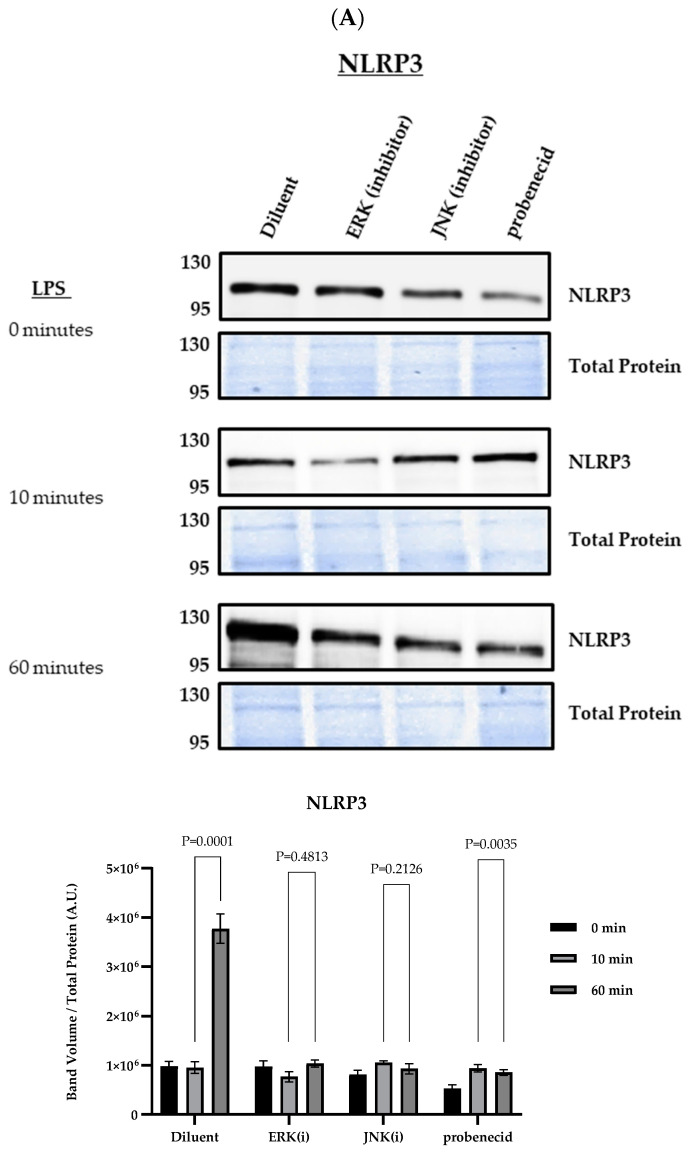

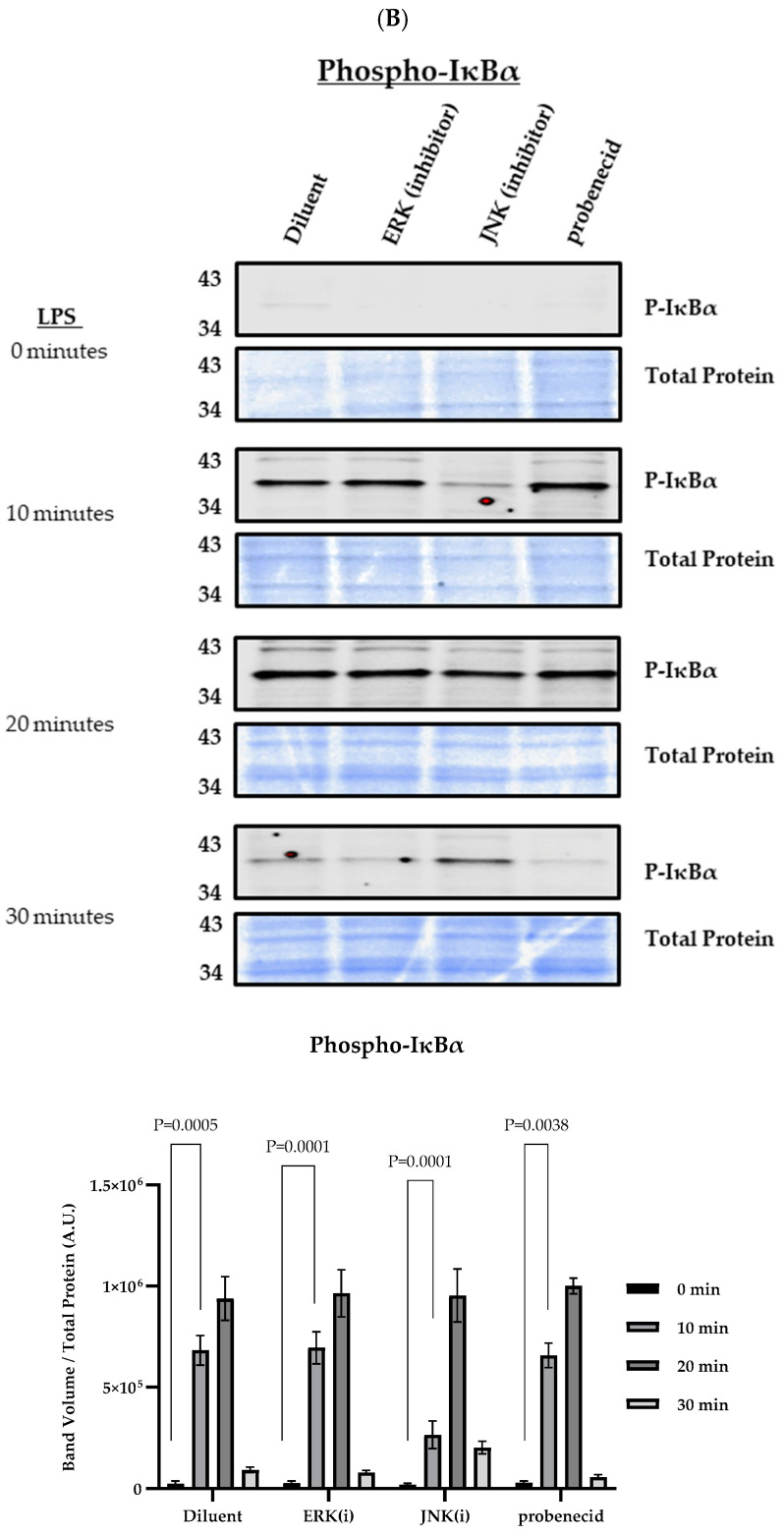
NF-κB-mediated protein expression in LPS-primed macrophages. J774A.1 macrophages were treated with 0.1 µM probenecid, 25 µM JNK inhibitor (SP600125), 10 µM ERK inhibitor (PD98059), or diluent (0.1% DMSO) for 2 h and stimulated with 100 ng/mL LPS from *E. coli* O111:B4 for 0, 10, or 60 min, or stimulated with 100 ng/mL LPS for 0, 10, 20, and 30 min. Western blots were performed using equal amounts of cell lysates (20 µL) separated on 4–20% gradient SDS-PAGE gels and then transferred to nitrocellulose membrane for immunoprobing. Membranes were probed for (**A**) the presence of NLRP3 proteins using anti-NLRP3 antibody (Cell Signaling) or for (**B**) phospho-IκBα proteins using anti-phospho-IκBα antibodies (Thermo Fisher). After 3x washes, the membranes were developed with SuperSignal West Pico PLUS chemiluminescent substrate (Thermo Fisher). Total protein staining of transferred membranes utilizing the fluorescent ‘no-stain’ protein labeling reagent (Thermo Fisher) was used to normalize for protein loading with (n = 3) independent replicates for each condition. Immunoblot and total protein blot images were obtained using the iBright 1500 Imager (Thermo Fisher), representing three independent replicates per condition. Background-corrected band volumes were normalized to total protein, and the mean was calculated from three independent replicates for each condition using iBright analysis software (Thermo Fisher). The graphs depict the band volume quantification in arbitrary units (A.U.) represented as mean ± SD. Original western blots can be found at Supplementary Materials.

The first (or priming) signal for NLRP3 inflammasome activation is initiated by the activation of TLRs, leading to MAPK signaling and the upregulation of NLRP3, pro-IL-1β, and pro-IL-18 gene expression through the NF-κB pathway, which is necessary for subsequent inflammasome assembly and activation by a second signal [45,46]. To determine the effect of probenecid on the first NLRP3 inflammasome activation signal, we examined the levels of NLRP3 protein expression in macrophages treated for 2 h with 0.1 μM probenecid, ERK inhibitor (10 μM PD98059), JNK inhibitor (25 μM SP600125), or diluent (0.1% DMSO), then primed with LPS (Figure 2). At 60 min post-LPS stimulation, the probenecid treatment lowered NLRP3 levels compared to the diluent-treated macrophages by >60%, and the treatment with the MAPK inhibitors, i.e., ERK and JNK, also led to similarly lower levels of NLRP3 protein compared to the diluent control (Figure 2A). It is known that continuous JNK-directed phosphorylation of NLRP3 protein is necessary to maintain NLRP3 protein stability [47]. Thus, NF-κB activation was evaluated by determining the phospho-IκBα protein following the probenecid treatment and LPS stimulation. The presence of the phospho-IκBα protein indicates NF-κB activation or the relief of negative regulation by the non-phosphorylated IκBα protein [48]. The results suggest that the probenecid treatment did not affect phospho-IκBα linked to the NF-κB activation (Figure 2B). Intriguingly, only the JNK inhibitor (25 μM SP600125) but not the ERK inhibitor (10 μM PD98059) negatively affected phospho-IκBα and the activation of NF-κB compared to the diluent control treatment at 10 min after LPS exposure (Figure 2B). However, this effect was reversed 20 min after the LPS stimulation, where the phospho-IκBα levels were similar to the diluent treatment, indicating NF-κB activation in the presence of the JNK inhibitor (Figure 2B). After 30 min of LPS stimulation, most phospho-IκBα protein was degraded via ubiquitin-mediated proteolysis through NF-kB regulation regardless of drug treatment (Figure 2B).

**Figure 3:**
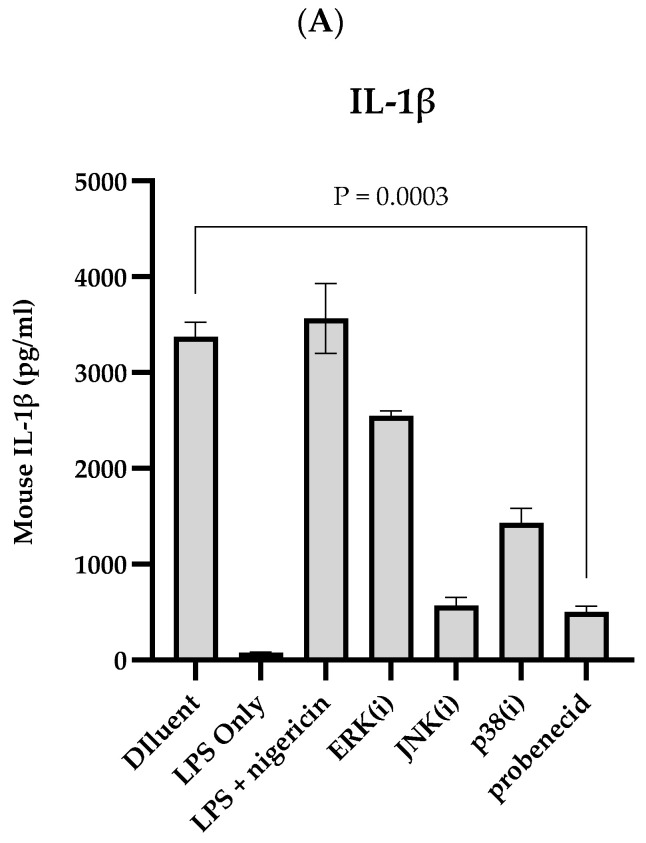

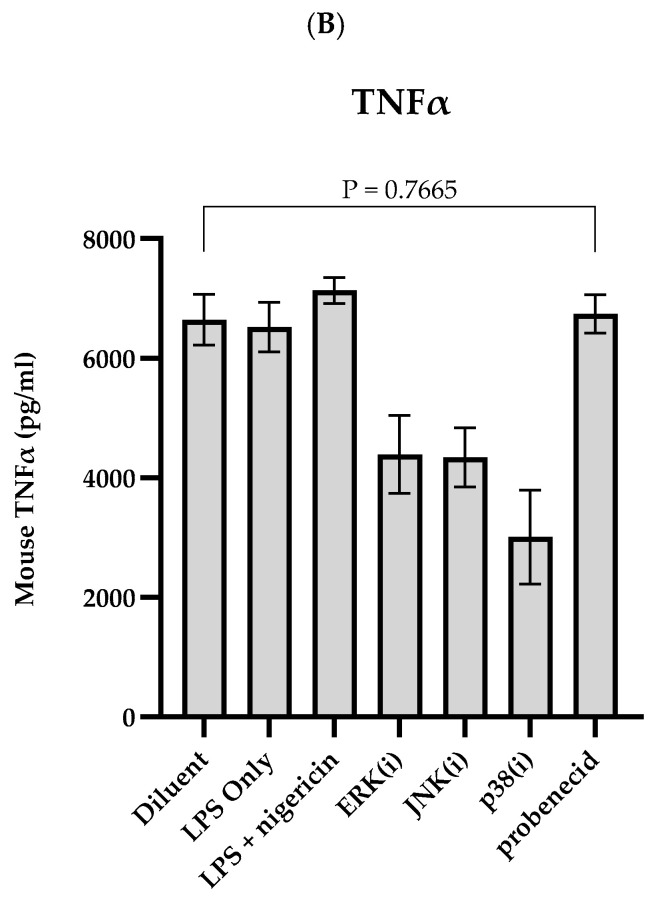
MAPK signaling and NLRP3 inflammasome activity in LPS and nigericin-activated macrophages. J774A.1 macrophages were stimulated with 100 ng/mL LPS for 6 h and 0.1 µM probenecid, 25 µM JNK inhibitor (SP600125), 10 µM ERK inhibitor (PD98059), 20 µM p38 inhibitor (SB202190), or diluent (0.1% DMSO) were added for the last 2 h of LPS treatment. NLRP3 inflammasome was activated by adding 20 µM nigericin for 1 h. LPS-primed macrophages without further treatment were activated with nigericin (LPS + nigericin) or not activated (LPS only) to serve as NLRP3 inflammasome activation controls. Culture supernatants were harvested and adjusted with 2 mM PMSF protease inhibitor. The cell-free supernatants were evaluated using a capture ELISA to measure the levels of (**A**) IL-1β protein, or (**B**) TNFα per the manufacturer’s instructions. Protein concentration was calculated from three independent replicates, represented as mean ± SD for each condition.

The effect of probenecid treatment and MAPK inhibitors on canonical NLRP3 inflammasome activation was investigated in the stimulated J774A.1 macrophages (Figure 3). The LPS-primed macrophages were treated with 0.1 μM probenecid, 10 μM ERK inhibitor (PD98059), 25 μM JNK inhibitor (SP600125), 20 μM p38 inhibitor (SB202190), or diluent (0.1% DMSO). Subsequently, the NLRP3 inflammasome was activated by adding 20 μM nigericin, and the cell-free culture supernatants were quantitated for IL-1β (Figure 3A) and TNFα (Figure 3B). As a control, LPS-primed macrophages without further treatment were activated by exposure to 20 μM nigericin or not activated with nigericin. The results suggest that IL-1β expression depends on nigericin-activated NLRP3 inflammasome activity and that the MAPK inhibitor treatments restrict IL-1β levels and NLRP3 inflammasome activation compared to the diluent-treated cells. The J774A.1 macrophages treated with 0.1 μM probenecid or 25 μM JNK inhibitor (SP600125) had >80% reduction in IL-1β upon NLRP3 inflammasome activation (Figure 3A). Although TNFα is released into the macrophage supernatants upon TLR ligation and NF-κB activation is independent of inflammasome activation, nigericin stimulation did not affect the TNFα levels in the LPS-primed J774A.1 macrophage supernatant. Treatment with the MAPK inhibitors exhibited a variable inhibition of TNFα after the inflammasome activation. However, the probenecid treatment did not affect the TNFα levels (Figure 3B), which is consistent with our previous results (Figure 2B), showing that probenecid does not affect NF-κB activation via the phospho-IκBα regulatory mechanism.

**Figure 4:**
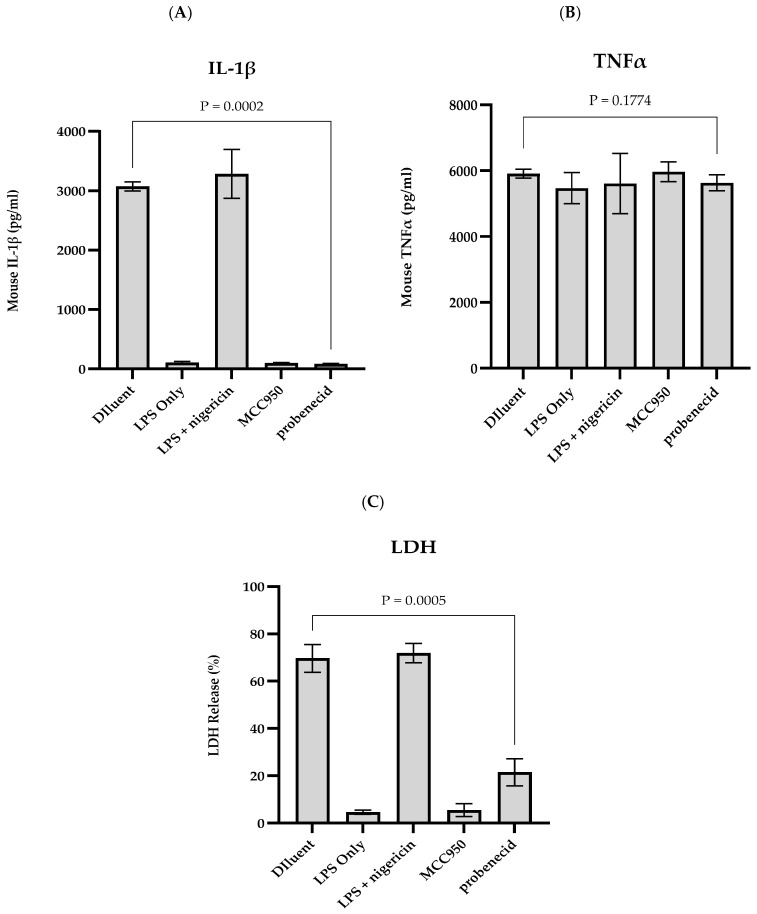
Probenecid treatment inhibits NLRP3 inflammasome activity in LPS and nigericin-activated macrophages. J774A.1 macrophage was stimulated with 100 ng/mL LPS for 6 h with the addition of 0.1 µM probenecid, 10 µM NLRP3 inhibitor (MCC950), or diluent (0.1% DMSO) for the last 2 h of LPS treatment. NLRP3 inflammasomes were activated by adding 20 µM nigericin for 1 h. LPS-primed macrophages without further treatment were activated with nigericin (LPS + nigericin) or not activated (LPS only) to serve as NLRP3 inflammasome activation controls. Culture supernatants were harvested and adjusted with 2 mM PMSF protease inhibitor. The cell-free supernatants were evaluated using a capture ELISA to measure the levels of (**A**) IL-1β protein, or (**B**) TNFα per the manufacturer’s instructions. Mean protein concentration was calculated from three independent replicates, represented as mean ± SD for each condition. (**C**) LDH assay for production of LDH per the manufacturer’s instructions. Data expressed as a percentage of maximal release calculated from three independent replicates per condition and represented as mean ± SD.

We sought to determine how probenecid treatment affected NLRP3 inflammasome activity in stimulated J774A.1 macrophages treated with 0.1 μM probenecid, 10 μM MCC950 (an NLRP3-specific inhibitor), or diluent control. MCC950 is a diaryl sulfonylurea compound that binds to the walker B motif of the NATCH domain vital for NLRP3 self-association and function [20]. LPS-primed J774A.1 macrophages treated with 0 or 20 μM nigericin were used as a control. The results show that IL-1β in the J774A.1 macrophage supernatant is dependent on nigericin-activated NLRP3 inflammasome activity, and treatment with the NLRP3-specific inhibitor (i.e., 10 μM MCC950) or 0.1 μM probenecid prevents IL-1β expression by >90% from the stimulated macrophages compared to the diluent-treated controls (Figure 4A). In addition, the TNFα expression in the culture supernatants is independent of the inflammasome activation by nigericin and was not affected by the MCC950 or probenecid treatment compared to the diluent-treated macrophages (Figure 4B). Notably, the LPS- and nigericin-activation of the NLRP3 inflammasomes leads to pyroptotic cell death, as measured by LDH release into the culture supernatants (Figure 4C). It is known that MCC950 is a potent inhibitor of pyroptosis, and notably, our results show that probenecid treatment can inhibit pyroptosis 3–4-fold after NLRP3 inflammasome activation (Figure 4C).

**Figure 5:**
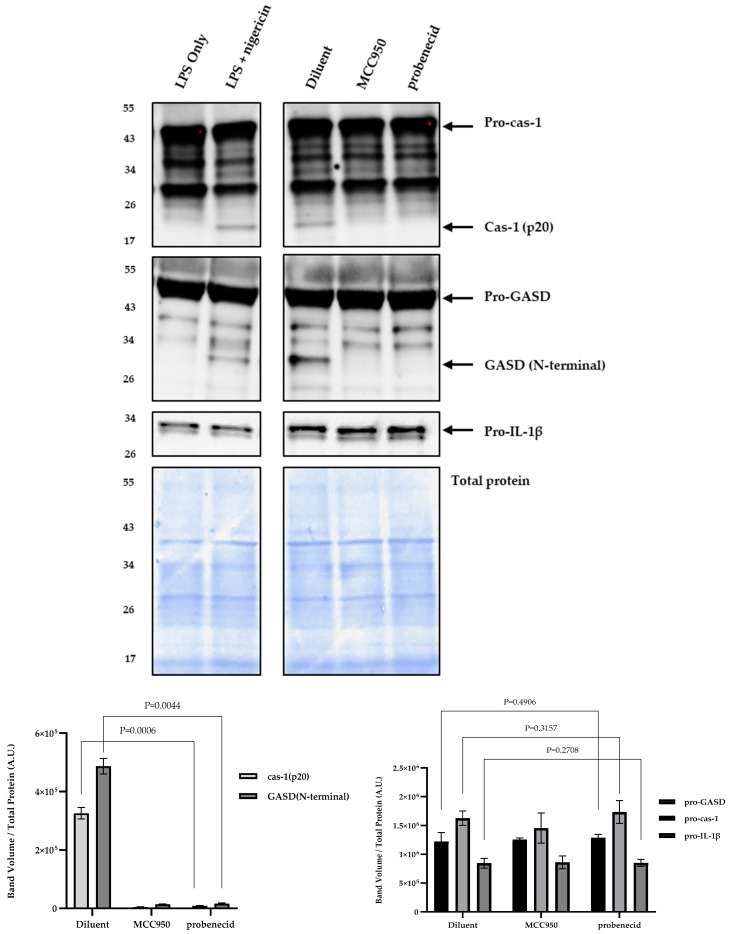
Probenecid treatment inhibits cas-1 and GASD in response to NLRP3 inflammasome activation in macrophages. J774A.1 macrophages were stimulated with 100 ng/mL LPS for 6 h with the addition of 0.1 µM probenecid, 10 µM NLRP3 inhibitor (MCC950), or diluent (0.1% DMSO) for the last 2 h of LPS treatment. The NLRP3 inflammasome was activated by adding 20 µM nigericin for 1 h. LPS-primed macrophages without further treatment were activated with nigericin (LPS + nigericin) or not activated (LPS only) to serve as NLRP3 inflammasome activation controls. Western blots were performed using equal amounts (20 µL) of a 1:1 mixture of cell lysates and culture supernatants separated on 4–20% gradient SDS-PAGE gels and then transferred to nitrocellulose membrane for immunoprobing. Membranes were detected using anti-cas-1 rabbit mAb (Cell Signaling), anti-cas-1 (p20 subunit) (Casper-1 Adipogen), anti-GASD rabbit mAb (Cell Signaling), anti-cleaved GASD (Asp276) rabbit mAb (Cell Signaling), or anti-IL-1β rabbit mAb (Cell Signaling). After three washes, the membrane signal was developed with SuperSignal West Pico PLUS chemiluminescent substrate (Thermo Fisher). Total protein staining of transferred membranes utilizing the fluorescent ‘no-stain’ protein labeling reagent (ThermoFisher) was used to normalize for protein loading with (n = 3) independent replicates for each condition. Immunoblot and total protein blot images were obtained using the iBright 1500 Imager (ThermoFisher), representing three independent replicates per condition. Background-corrected band volumes were normalized to total protein, and the mean was calculated from three independent replicates for each condition using iBright analysis software (ThermoFisher). The graphs depict the band volume quantification in arbitrary units (A.U.) represented as mean ± SD. Original western blots can be found at Supplementary Materials.

Consistent with the findings of this study that neither MCC950 nor probenecid affects TNFα expression (Figure 4B), we show that stimulated J774A.1 macrophages treated with 10 μM MCC950, 0.1 μM probenecid, or diluent have similar levels of pro-cas-1, pro-GASD, and pro-IL-1β proteins upon NLRP3 inflammasome activation by LPS and nigericin (Figure 5). In contrast, this study shows that IL-1β levels are inhibited by >90% following MCC950 or probenecid treatment compared to the diluent control during NLRP3 inflammasome activation (Figure 4A). The pro-IL-1β protein requires proteolytic processing by cas-1 (p20) to IL-1β upon NLRP3 inflammasome activation [49,50]. Interestingly, MCC950 or probenecid treatment results in the loss of detectable cas-1 (p20) compared to the diluent in the stimulated macrophages undergoing NLRP3 inflammasome activation (Figure 5). In addition, treating these cells with MCC950 or probenecid also resulted in the complete loss of detectable cleaved GASD (N-terminal) compared to diluent treatment (Figure 5). LPS-primed cells without further treatment were activated by exposure to 0 or 20 μM nigericin treatment (Figure 5). The results show that pro-cas-1 proteolytic processing to cas-1 and subsequent cas-1-directed proteolytic cleavage of both pro-IL-1β and pro-GASD to their mature forms are dependent upon nigericin activation of the NLRP3 inflammasome (Figure 5). The observation that probenecid treatment of LPS-primed and nigericin-activated macrophages leads to the loss of detectable cas-1 is consistent with our finding that probenecid treatment results in both the inhibition of IL-1β protein and the complete loss of detectable mature GASD (N-terminal) because both pro-GASD and pro-IL-1β require cas-1 to activate canonical NLRP3 inflammasomes.

## 4. Discussion

The NLRP3 inflammasome has a fundamental role in the inflammatory response. Upon activation, the NLRP3 inflammasome triggers a cascade of reactions, including the release of activated cas-1, which causes the expression of IL-1β and other pro-inflammatory cytokines, amplifying the inflammatory response [18]. The exposure of macrophages to LPS constitutes the first of two signals required for the canonical activation of the NLRP3 inflammasome, the second signal being K+ ion efflux from cells initiated by the bacterial toxin nigericin, ROS, or other stress-related stimuli, e.g., virus infection [18,51]. In the canonical NLRP3 activation model, LPS stimulation (first signal) activates NF-κB-directed gene expression and protein translation to generate the NLRP3 inflammasome-associated pro-proteins, i.e., pro-IL-1β, pro-cas-1, and pro-GASD [18]. These pro-proteins require further post-translational processing to their mature active forms to achieve NLRP3 inflammasome activation. Post-translational processing of the various NLRP3 inflammasome pro-proteins constitutes an additional level of control of NLRP3 inflammasome activation, separate from the first activation signal initiated by the second activation signal [52].

Measuring the IL-1β produced by macrophages can be used as an indicator of NLRP3 inflammasome activation [53]. We tested the pharmacological activation inhibitors of ERK, JNK, and p38 MAPKs to determine the effects of MAPK signaling on the NLRP3 inflammasome activation induced by LPS and nigericin treatment. We showed that JNK signaling is necessary for NLRP3 inflammasome activation in the macrophages and that probenecid inhibits ERK and JNK-directed MAPK signaling but not p38 MAPK signaling, resulting in decreased levels of NLRP3 protein in response to LPS stimulation. Indeed, studies have shown that the JNK-mediated phosphorylation, not the NF-κB-directed gene transcription of NLRP3, is essential for rapid and sustained NLRP3 inflammasome activation. While NF-κB activation is not necessary for inflammasome responses, the elevated expression of NLRP3 and pro-proteins by NF-κB contribute to the robustness of the reaction. Moreover, the activation of the TLR-JNK1 axis by 10 min of LPS exposure is a necessary priming event for the activation and stabilization of NLRP3 inflammasome as the JNK1-mediated phosphorylation of NLRP3 at S194 prevents NLRP3 ubiquitination and proteolysis [47]. Thus, blocking JNK activity early upon inflammatory stimulus results in a rapid turnover of NLRP3 proteins and a reduced NLRP3 inflammasome response in J774A.1 macrophages. It is not clear why phospho-p38 levels appear to be unaffected by probenecid, but studies with MAPK inhibitors examining phosphorylation of p38, ERK1/2, and JNK1/2 in IL-1β-induced signaling show that JNK inhibition significantly attenuates ERK1/2 phosphorylation but not p38 phosphorylation [1]. Moreover, JNK1/2 and ERK1/2 are co-immunoprecipitated with a phospho-JNK specific antibody, suggesting that p38 signaling is independently activated while JNK signaling interacts with ERK1/2 signaling [54]. Probenecid inhibits JNK signaling [1] and is more likely to affect ERK1/2 signaling but not p38.

We show that probenecid inhibits IL-1β expression and pyroptosis in macrophages but not TNFα following LPS and nigericin activation of NLRP3 inflammasomes. Previously, studies have shown that probenecid can inhibit the AIM2 inflammasome in vitro in human cell lines activated with liposome-encapsulated poly(dA:dT), a specific AIM2 inflammasome inducer [55]. Co-immunoprecipitation of AIM2 and pannexin-1 hemichannel protein from activated cells suggests that the purinergic receptor, P2X7R, and pannexin-1 are involved in AIM2 activation, offering a plausible mechanism for probenecid’s inhibitory effects on AIM2 activation by blocking the pannexin-1 hemichannel. Other studies have shown that pannexin-1 is dispensable for NLRP3 activation [29]. Although many NLRP3 agonists are associated with membrane channel opening, this is insufficient for NLRP3 activation, given the requirement for potassium efflux. In this study, probenecid treatment abrogated cas-1 auto-proteolysis in NLRP3 inflammasome-activated macrophages. Additionally, probenecid treatment inhibited cas-1-dependent GASD maturation. The JNK-mediated phosphorylation of NLRP3 prevents its ubiquitination and proteolysis [47]. Without competent JNK activity, NLRP3 inflammasome assembly, stability, and the ability to recruit caspase-1 via the ASC adaptor protein are negatively impacted. Probenecid, through its inhibitory effects on JNK activity, may negatively affect the stability and function of NLRP3 inflammasomes.

## 5. Conclusions

We show that probenecid treatment does not affect NF-κB-dependent protein expression levels of pro-cas-1, pro-GASD, and pro-IL-1β. Our observations suggest that probenecid inhibits the processing pro-cas-1 to mature catalytically active cas-1 (p20), which is necessary for IL-1β and GASD. Thus, probenecid affects the NLRP3 inflammasome response in a mouse macrophage cell line after the cells sense the second activation signal, i.e., the potassium efflux initiated by nigericin. We are currently determining if these effects in a mouse model of NLRP3 inflammasome activation translate to human cells. These findings in the mouse model represent a unique mechanism of action of probenecid, separate from its known ability to block pannexin 1 channels after purinergic receptor ligation in connection with NLRP3 inflammasome activity.

## Data Availability

The data supporting the reported results are in the Tripp laboratory at the University of Georgia.

## Supplementary Materials

The following supporting information can be downloaded at: https://www.mdpi.com/article/10.3390/biom15040511/s1, Figure S1. MAPK signaling in LPS-primed macrophages. Figure S2. NF-κB-mediated protein expression in LPS-primed macrophages. Figure S3. MAPK signaling and NLRP3 inflammasome activity in LPS and nigericin-activated macrophages. Figure S4. Probenecid treatment inhibits NLRP3 inflammasome activity in LPS and nigericin-activated macrophages. Figure S5. Probenecid treatment inhibits cas-1 and GASD in response to NLRP3 inflammasome activation in macrophages. File S1. Original WB.
