# Supplementary material for: Probenecid Inhibits NLRP3 Inflammasome Activity and Mitogen-Activated Protein Kinases (MAPKs)
Source: Biomolecules. 2025 Apr 1;15(4):511. doi: 10.3390/biom15040511 (PMC12024562; doi:10.3390/biom15040511)
Supplement: Supplementary file 1 [file biomolecules-15-00511-s001.zip › biomolecules-3481820-Supplementary File S1-original WB.pdf]

Supplementary Material

Figure S1A. MAPK signaling in LPS-primed macrophages

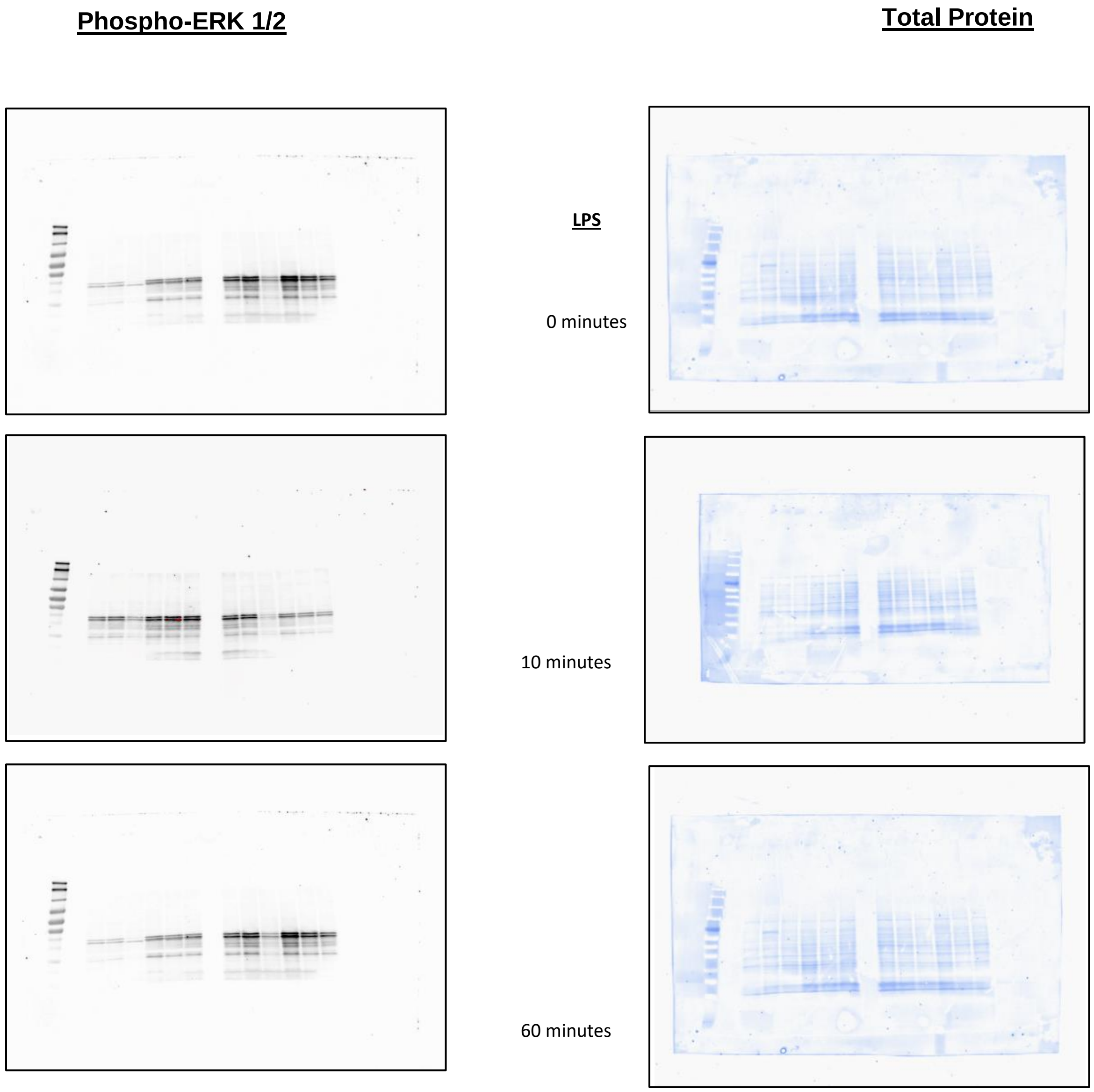

*TPN Band Volume	0 min			10 min			60 min		
**P-ERK 1/2**	**Mean**	**SD**	**N**	**Mean**	**SD**	**N**	**Mean**	**SD**	**N**
**Diluent**	216667	17954	3	1443333	81445	3	1700000	238118	**3**
**ERK(i)**	105667	5686	3		57117	3	403667	60003	**3**
**JNK(i)**	408000	53000	3	501667	77397	3	2906667	577610	**3**
**probenecid**	438000	42036	3	443667	78053	3	1310000	185203	**3**

*Total protein normalized band volumes

Figure S1B.

Phospho-JNK 1/2 **Total Protein**

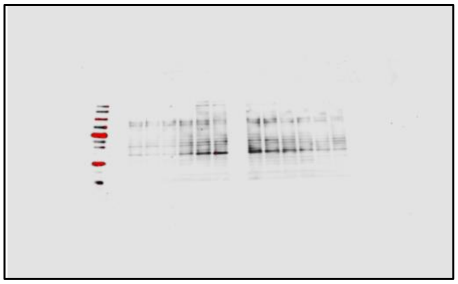

LPS

0 minutes

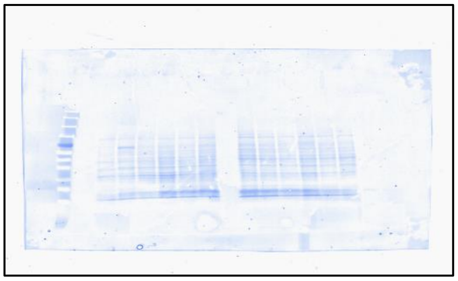

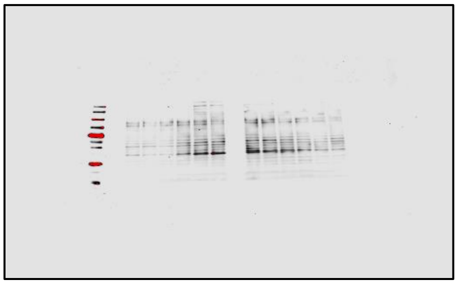

10 minutes

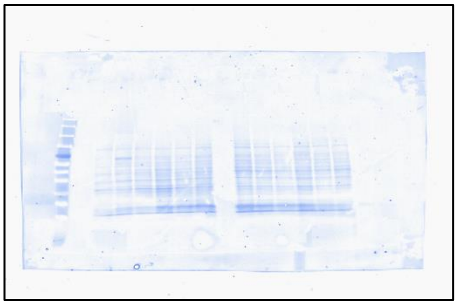

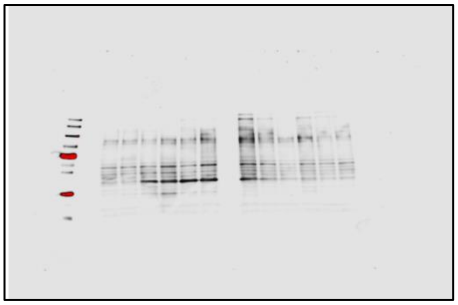

60 minutes

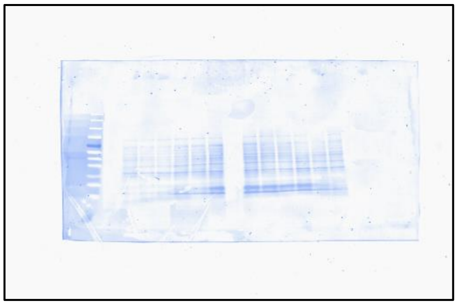

*TPN Band Volume	0 min			10 min			60 min		
**P-JNK 1/2**	**Mean**	**SD**	**N**	**Mean**	**SD**	**N**	**Mean**	**SD**	**N**
**Diluent**	47683	6313	3	1658090	217047	3	42922	15990	3
**ERK(i)**	55037	5519	3	1165919	118292	3	46456	21907	3
**JNK(i)**	53874	4276	3	276804	67460	3	25325	10083	3
**probenecid**	108163	9054	3	144982	34227	3	34159	5688	3

*Total protein normalized band volumes

Figure S1C.

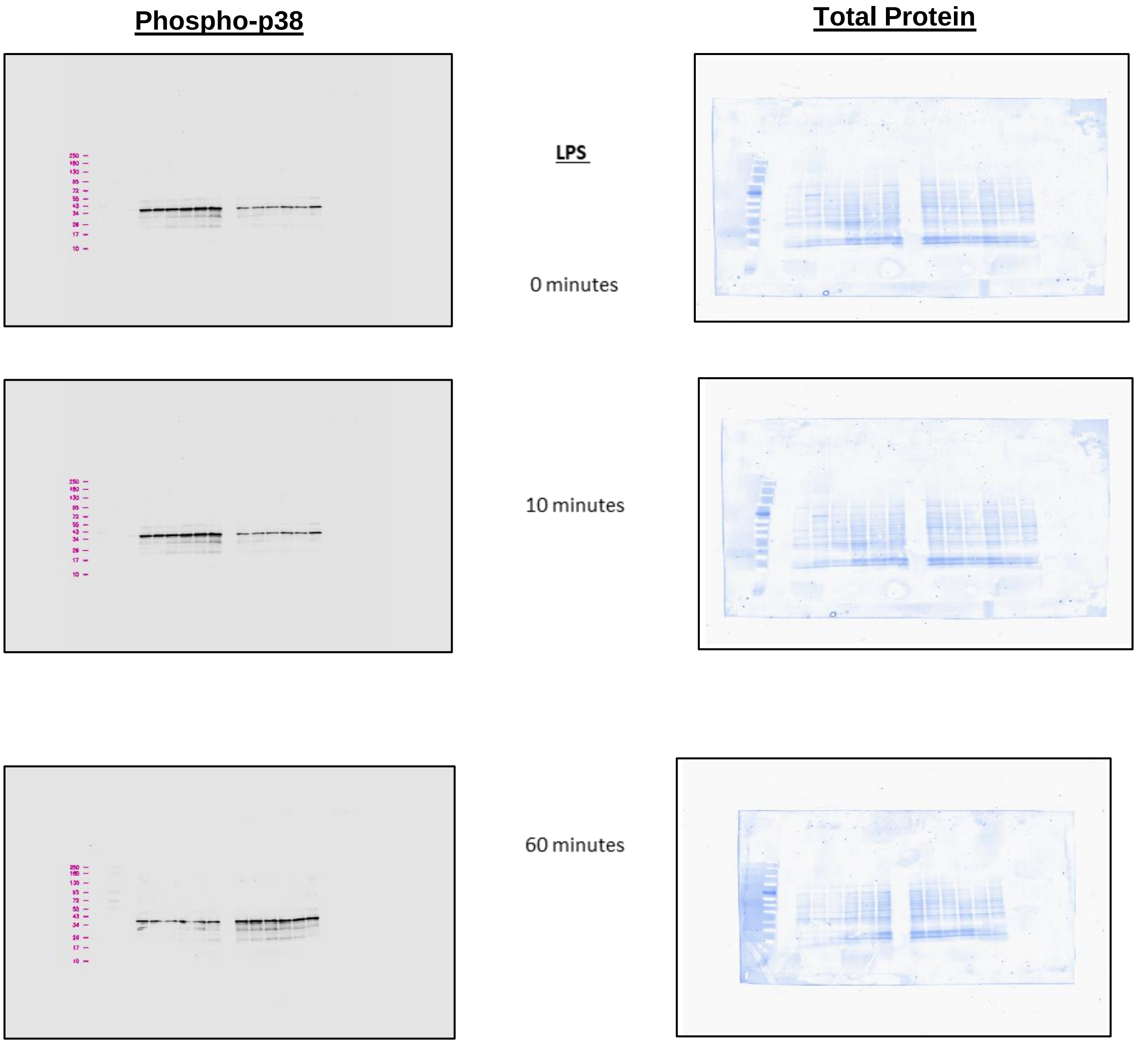

*TPN Band Volume	**0 min**			**10 min**			**60 min**		
**P-p38**	**Mean**	**SD**	**N**	**Mean**	**SD**	**N**	**Mean**	**SD**	**N**
**Diluent**	747667	119509	3	1406667	370045	3	288333	13503	3
**ERK(i)**	564667	144362	3	1330000	229129	3	261333	22723	3
**JNK(i)**	662667	220012	3	1400000	174356	3	243667	50639	3
**probenecid**	665333	281727	3	1363333	111505	3	263333	61158	3

*Total protein normalized band volumes

Figure S2A. NFκB-mediated protein expression in LPS-primed macrophages.

NLRP3 | **Total Protein**

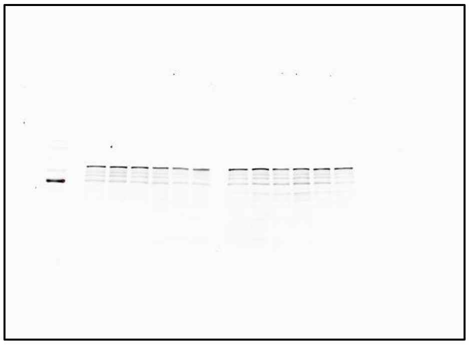

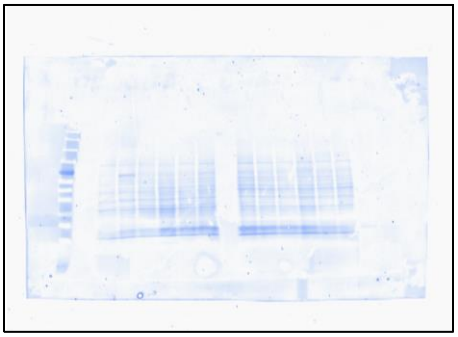

LPS

0 minutes

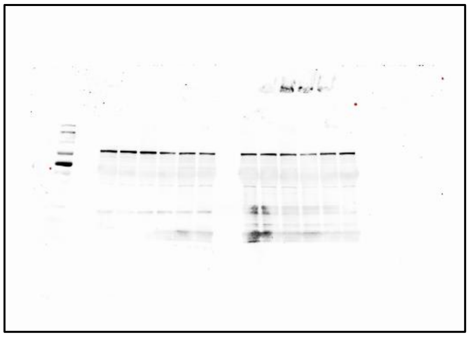

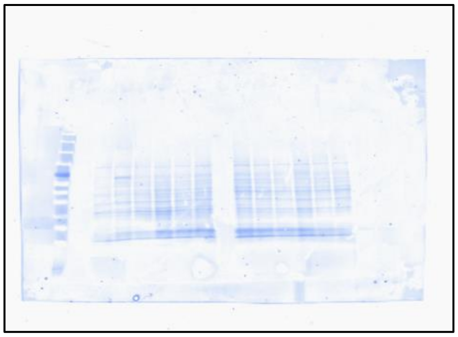

10 minutes

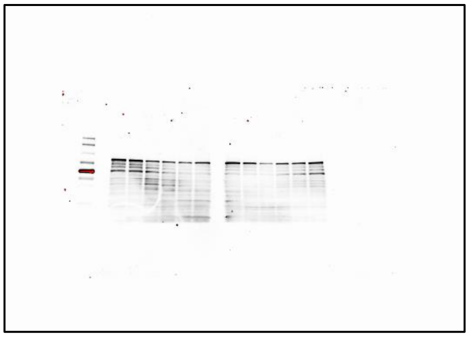

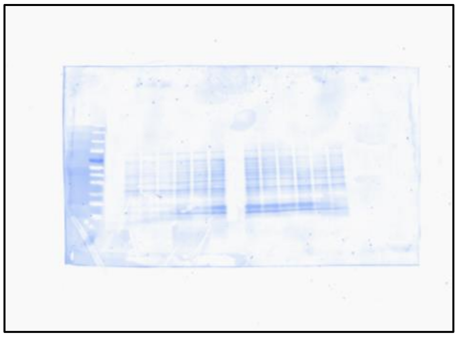

60 minutes

*TPN Band Volume	0 min			10 min			60 min		
**NLRP3**	**Mean**	**SD**	**N**	**Mean**	**SD**	**N**	**Mean**	**SD**	**N**
**Diluent**	979582	100747	3	952930	117921	3	3771059	300195	3
**ERK(i)**	974368	113132	3	767958	106591	3	1034296	71556	3
**JNK(i)**	810879	91963	3	1054797	35725	3	931122	106357	3
**probenecid**	526959	74557	3	937920	74909	3	854922	55078	3

*Total protein normalized band volumes

Figure S2B.

Phospho-IkBα **Total Protein**

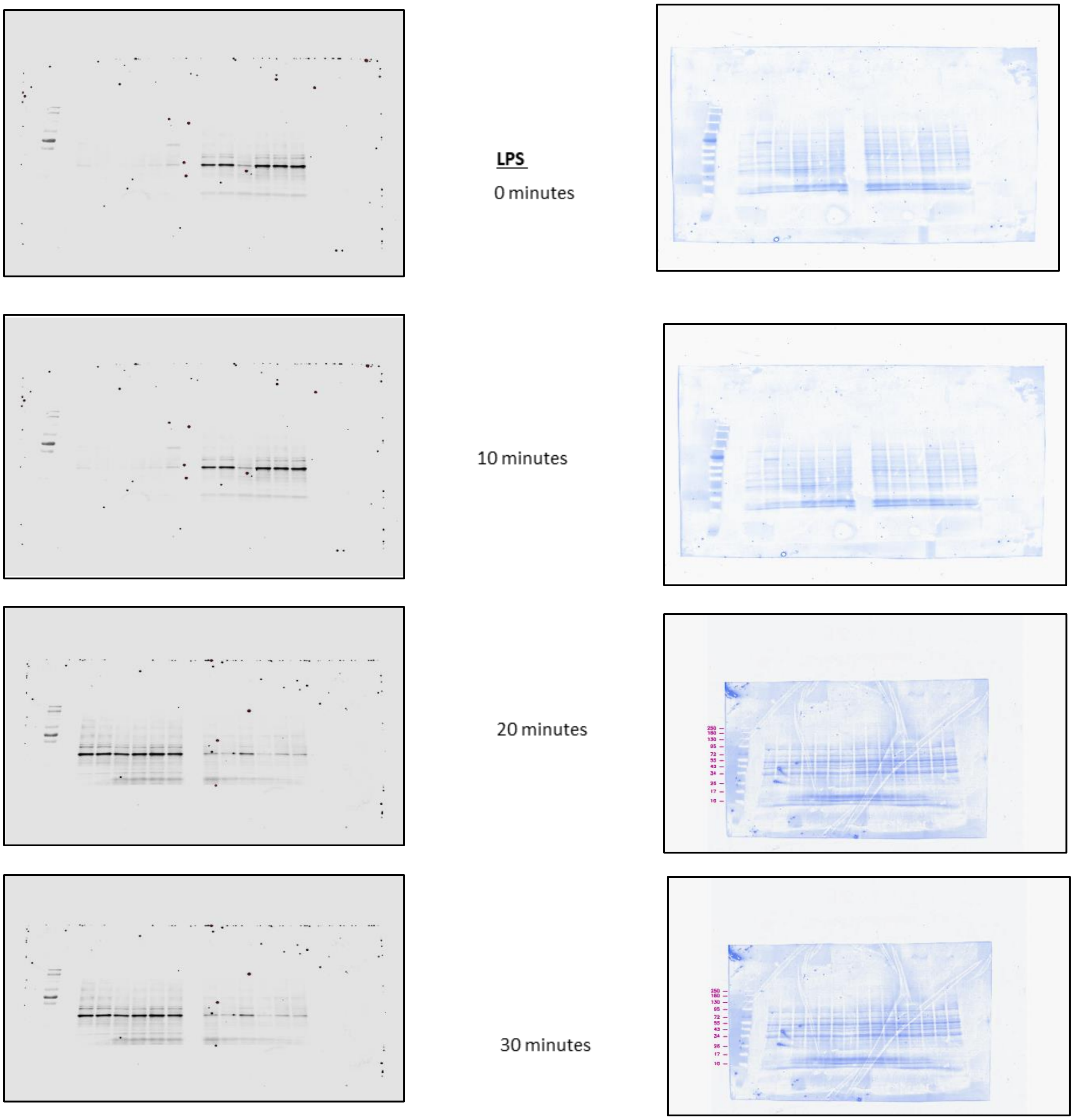

*TPN Band Volume	0 min			10 min			20 min			30 min		
**P-IκBα**	**Mean**	**SD**	**N**	**Mean**	**SD**	**N**	**Mean**	**SD**	**N**	**Mean**	**SD**	**N**
**Diluent**	24473	12939	3	682606	72778	3	938400	107938	3	91828	14284	3
**ERK(i)**	27308	8976	3	695563	79420	3	964550	116287	3	78389	10518	3
**JNK(i)**	18812	7058	3	264866	68056	3	953517	130319	3	201900	31604	3
**probenecid**	27857	8531	3	657474	60878	3	1000624	38404	3	56010	12169	3

*Total protein normalized band volumes

Figure S5. Probenecid treatment inhibits cas-1 and GASD in response to NLRP3 inflammasome activation in macrophages

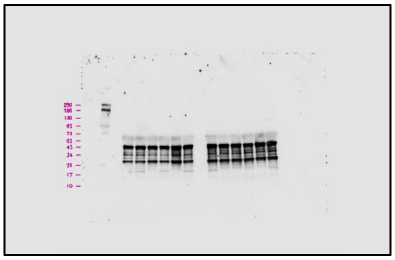

Pro-cas-1
Cas-1(p20)

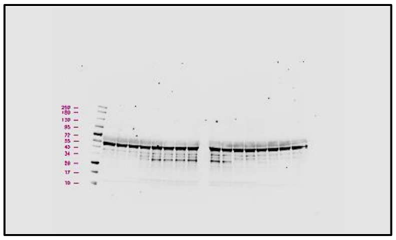

Pro-GASD and
GASD (NT)

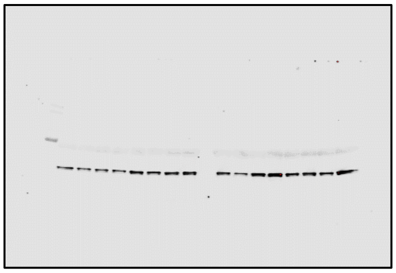

Pro-IL-1β

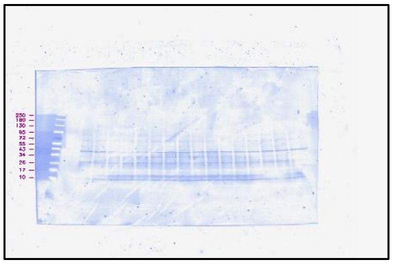

Total Protein

*TPN Band Volume	**cas-1 (p20)**			**GASD (N-Terminal)**			**pro-cas-1**			**pro-GASD**			**pro-IL-1β**		
	**Mean**	**SD**	**N**	**Mean**	**SD**	**N**	**Mean**	**SD**	**N**	**Mean**	**SD**	**N**	**Mean**	**SD**	**N**
**Diluent**	325724	19880	3	486827	27123	3	1221306	157058	3	1626408	125100	3	844212	85163	3
**MCC950**	4397	1135	3	13824	1505	3	1255045	28989	3	1455826	262412	3	860677	111829	3
**probenecid**	8321	1274	3	15718	2732	3	1286683	60482	3	1734193	197606	3	849616	62100	3

*Total protein normalized band volumes
