# Supplementary material for: Probenecid Inhibits NLRP3 Inflammasome Activity and Mitogen-Activated Protein Kinases (MAPKs)
Source: Biomolecules. 2025 Apr 1;15(4):511. doi: 10.3390/biom15040511 (PMC12024562; doi:10.3390/biom15040511)
Supplement: Supplementary file 1 [file biomolecules-15-00511-s001.zip › biomolecules-3481820-supplementary.pdf]

Supplementary Figure S1. MAPK signaling in LPS-primed macrophages.

A

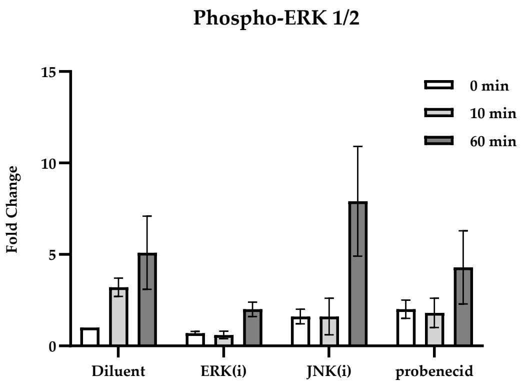

B

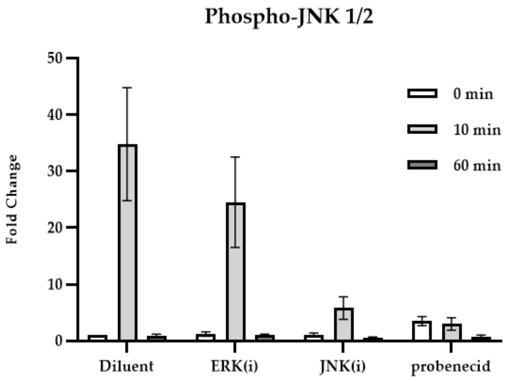

C

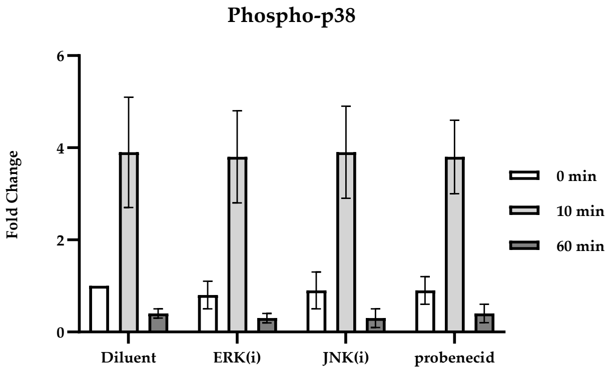

Legend Supplementary Figure S1. J774A.1 macrophages were treated with 0.1 μM probenecid, 25 μM JNK inhibitor (SP600125), 10 μM ERK inhibitor (PD98059), or diluent (0.1% DMSO) as described. Samples were probed for phospho-ERK1/2 proteins using anti-phospho-p44/42 MAPK (ERK1/2) (Thr202/Tyr204), phospho-JNK1/2 proteins using anti-phospho-SAPK/JNK (Thr183/Tyr185), or phospho-p38 proteins using anti-phospho-p38 MAPK (Thr180/Tyr182) (Cell Signaling). The membranes were washed three times with TBS-T, and the signal developed with SuperSignal West Pico PLUS chemiluminescent substrate (Thermo Fisher). Total protein staining of transferred membranes utilizing the fluorescent 'no-stain' protein labeling reagent (Thermo Fisher) was used to normalize for protein loading with (n = 3) independent replicates for each condition. Immunoblot and total protein blot images were obtained using the iBright 1500 Imager (Thermo Fisher), representing three independent replicates per condition. Background-corrected band volumes were normalized to total protein, and the mean was calculated from three independent replicates for each condition using iBright analysis software (Thermo Fisher). The graphs depict the fold-change in mean band volume after treatment for **A**. phospho-ERK1/2, **B**. phospho-JNK1/2, or C. phospho-p38 proteins relative to diluent treatment (0 minutes) condition, represented as mean ± SEM.

Supplementary Figure S2. NF-κB-mediated protein expression in LPS-primed macrophages.

A

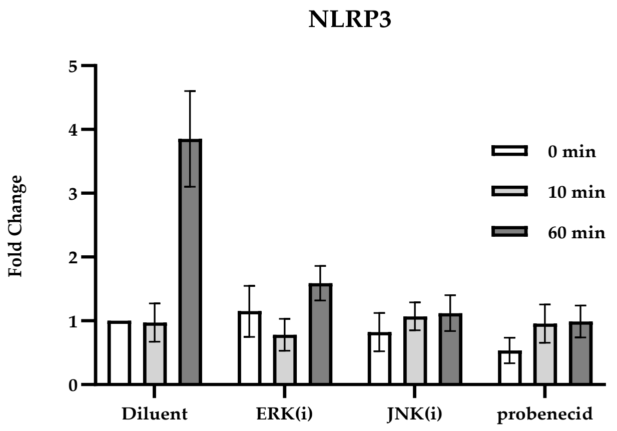

B

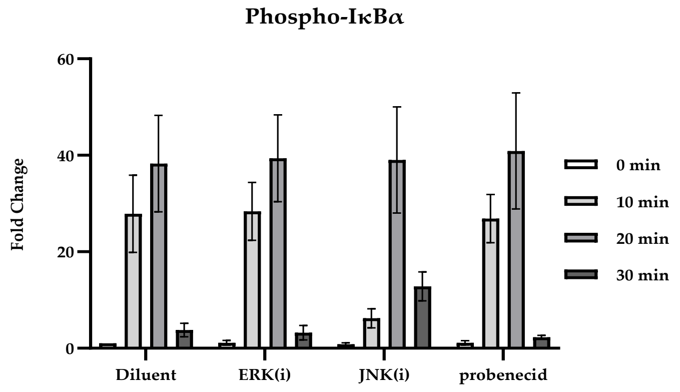

Legend Supplementary Figure S2. J774A.1 macrophages were treated with 0.1 μM probenecid, 25 μM JNK inhibitor (SP600125), 10 μM ERK inhibitor (PD98059), or diluent (0.1% DMSO) for 2 h and stimulated with 100 ng/ml LPS from *E. coli* O111:B4 for 0, 10, or 60 min, or stimulated with 100 ng/ml LPS for 0, 10, 20, and 30 min. Western blots were performed using equal amounts of cell lysates (20 μl) separated on 4-20% gradient SDS-PAGE gels and then transferred to nitrocellulose membrane for immunoprobing. Membranes were probed for the presence of NLRP3 proteins using anti-NLRP3 antibody (Cell Signaling) or for phospho-IκBα proteins using anti-phospho-IκBα antibodies (Thermo Fisher). After 3x washes, the membranes were developed with SuperSignal West Pico PLUS chemiluminescent substrate (Thermo Fisher). Total protein staining of transferred membranes utilizing the fluorescent 'no-stain' protein labeling reagent (Thermo Fisher) was used to normalize for protein loading with (n = 3) independent replicates for each condition. Immunoblot and total protein blot images were obtained using the iBright 1500 Imager (Thermo Fisher), representing three independent replicates per condition. Background-corrected band volumes were normalized to total protein, and the mean was calculated from three independent replicates for each condition using iBright analysis software (Thermo Fisher). The graphs depict the fold change in mean band volume after treatment for **A**. NLRP3, or **B**. phospho-IκBα proteins relative to diluent treatment (0 minutes) condition, represented as mean ± SEM.

Supplementary Figure S3. MAPK signaling and NLRP3 inflammasome activity in LPS and nigericin-activated macrophages

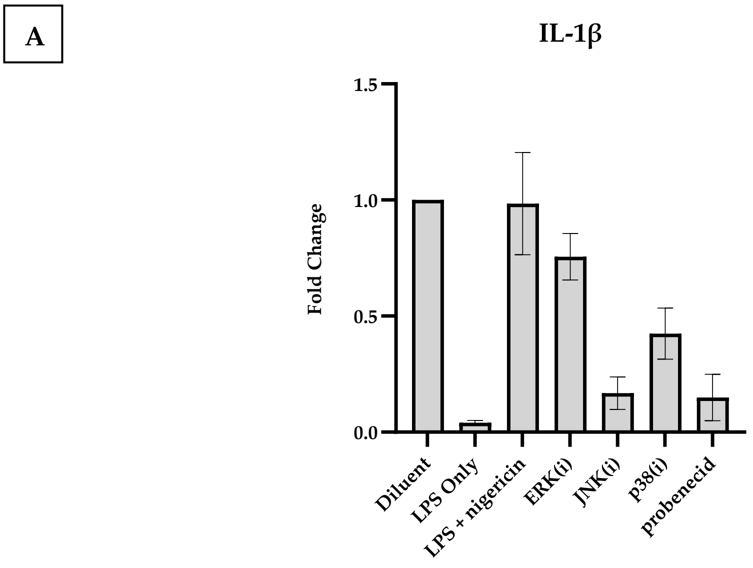

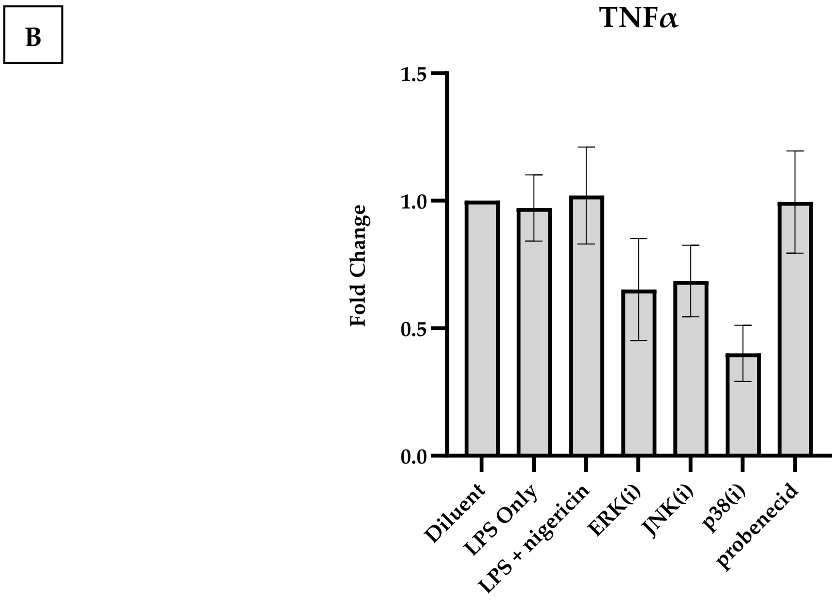

Legend Supplementary Figure S3. J774A.1 macrophages were stimulated with 100 ng/ml LPS for 6 h and 0.1 μM probenecid, 25 μM JNK inhibitor (SP600125), 10 μM ERK inhibitor (PD98059), 20 μM p38 inhibitor (SB202190), or diluent (0.1% DMSO) were added for the last 2 h of LPS treatment. NLRP3 inflammasome was activated by adding 20 μM nigericin for 1 h. LPS-primed macrophages without further treatment were activated with nigericin (LPS + nigericin), or not activated (LPS only) to serve as NLRP3 inflammasome activation controls. Culture supernatants were harvested and adjusted with 2 mM PMSF protease inhibitor. The cell-free supernatants were evaluated using a capture ELISA to measure the levels of IL-1β protein or TNFα per the manufacturer's instructions. Protein concentration was calculated from three independent replicates for each condition. Graphs depict the fold change after treatment in mean band volume of **A.** IL-1β, or **B**. TNFα proteins relative to the diluent treatment condition, represented as mean ± SEM.

Supplementary Figure S4. Probenecid treatment inhibits NLRP3 inflammasome activity in LPS and nigericin-activated macrophages

A

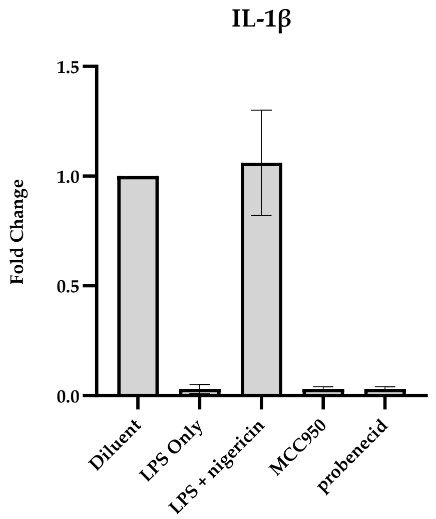

B

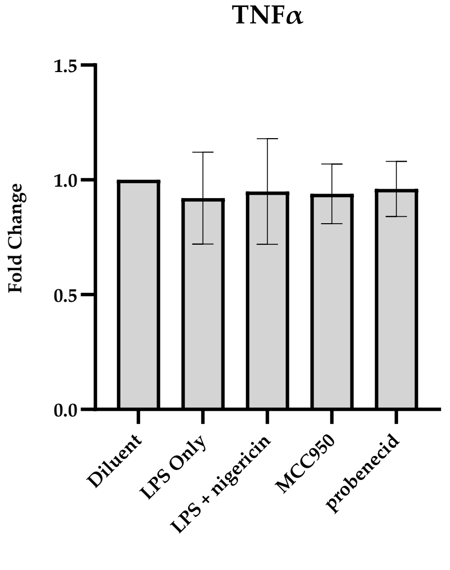

Legend Supplementary Figure S4. J774A.1 macrophages were stimulated with 100 ng/ml LPS for 6h with the addition of 0.1 μM probenecid, 10 μM NLRP3 inhibitor (MCC950), or diluent (0.1% DMSO) for the last 2 h of LPS treatment. NLRP3 inflammasomes were activated by adding 20 μM nigericin for 1 h. LPS-primed macrophages without further treatment were activated with nigericin (LPS + nigericin), or not activated (LPS only) to serve as NLRP3 inflammasome activation controls. Culture supernatants were harvested and adjusted with 2 mM PMSF protease inhibitor. The cell-free supernatants were evaluated using a capture ELISA to measure the levels of IL-1β or TNFα proteins per the manufacturer's instructions. Mean protein concentration was calculated from three independent replicates for each condition. Graphs depict the fold change after treatment in mean band volume of **A.** IL-1β, or **B**. TNFα proteins relative to the diluent treatment condition, represented as mean ± SEM.

Supplementary Figure S5. Probenecid treatment inhibits cas-1 and GASD in response to NLRP3 inflammasome activation in macrophages.

A

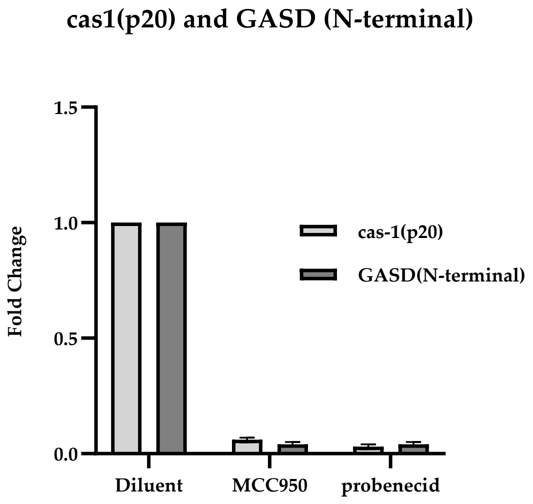

B

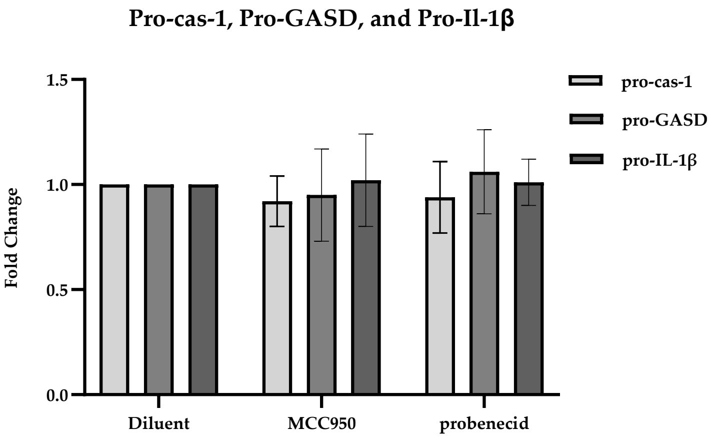

Legend Supplementary Figure S5. J774A.1 macrophages were stimulated with 100 ng/ml LPS for 6h with the addition of 0.1 μM probenecid, 10 μM NLRP3 inhibitor (MCC950), or diluent (0.1% DMSO) for the last 2 h of LPS treatment. The NLRP3 inflammasome was activated by adding 20 μM nigericin for 1 h. LPS-primed macrophages without further treatment were activated with nigericin (LPS + nigericin) or not activated (LPS only) to serve as NLRP3 inflammasome activation controls. Western blots were performed using equal amounts (20 μl) of a 1:1 mixture of cell lysates and culture supernatants separated on 4-20% gradient SDS-PAGE gels and then transferred to

nitrocellulose membrane for immunoprobing. Membranes were detected using anti-cas-1 rabbit mAb (Cell Signaling), anti-cas-1 (p20 subunit) (Casper-1, Adipogen), anti-GASD rabbit mAb (Cell Signaling), anti-cleaved GASD (Asp276) rabbit mAb (Cell Signaling), or anti-IL-1β rabbit mAb (Cell Signaling). After three washes, the membrane signal was developed with SuperSignal West Pico PLUS chemiluminescent substrate (Thermo Fisher). Total protein staining of transferred membranes utilizing the fluorescent 'no-stain' protein labeling reagent (ThermoFisher) was used to normalize for protein loading with (n = 3) independent replicates for each condition. Immunoblot and total protein blot images were obtained using the iBright 1500 Imager (ThermoFisher), representing three independent replicates per condition. Background-corrected band volumes were normalized to total protein, and the mean was calculated from three independent replicates for each condition using iBright analysis software (ThermoFisher). Graphs depict the fold change after treatment in mean band volume of **A.** cas-1 (p20) and GASD (N-terminal) or **B.** Pro-cas-1, Pro-GASD, and Pro-IL-1β proteins relative to the diluent treatment condition, represented as mean ± SEM.
